# Inflammatory aging clock: A cancer clock to characterize the patients’ subtypes and predict the overall survival in glioblastoma

**DOI:** 10.3389/fgene.2022.925469

**Published:** 2022-08-11

**Authors:** Lei Zhu, Feng Wang, Jiannan Huang, He Wang, Guangxue Wang, Jianxin Jiang, Qinchuan Li

**Affiliations:** ^1^ Department of Thoracic Surgery, Shanghai Pulmonary Hospital, School of Medicine, Tongji University, Shanghai, China; ^2^ Department of Thoracic Surgery, Shanghai East Hospital, School of Medicine, Tongji University, Shanghai, China; ^3^ Department of Oncology, Chongqing General Hospital, University of Chinese Academy of Sciences, Chongqing, China; ^4^ Department of Neurosurgery, Jilin Province People’s Hospital, Changchun, China; ^5^ Department of Critical Care Medicine, Shanghai East Hospital, School of Medicine, Tongji University, Shanghai, China; ^6^ Research Center for Translational Medicine, Shanghai East Hospital, Tongji University School of Medicine, Shanghai, China; ^7^ Department of Neurosurgery, Taizhou People’s Hospital Affiliated to Nanjing Medical School, Taizhou, China

**Keywords:** inflammatory age, chronological age, cancer clock, survival, glioblastoma, stem cell

## Abstract

**Background:** Many biological clocks related to aging have been linked to the development of cancer. A recent study has identified that the inflammatory aging clock was an excellent indicator to track multiple diseases. However, the role of the inflammatory aging clock in glioblastoma (GBM) remains to be explored. This study aimed to investigate the expression patterns and the prognostic values of inflammatory aging (iAge) in GBM, and its relations with stem cells.

**Methods:** Inflammation-related genes (IRG) and their relations with chronological age in normal samples from the Cancer Genome Atlas (TCGA) were identified by the Spearman correlation analysis. Then, we calculated the iAge and computed their correlations with chronological age in 168 patients with GBM. Next, iAge was applied to classify the patients into high- and low-iAge subtypes. Next, the survival analysis was performed. In addition, the correlations between iAge and stem cell indexes were evaluated. Finally, the results were validated in an external cohort.

**Results:** Thirty-eight IRG were significantly associated with chronological age (|coefficient| > 0.5), and were used to calculate the iAge. Correlation analysis showed that iAge was positively correlated with chronological age. Enrichment analysis demonstrated that iAge was highly associated with immune cells and inflammatory activities. Survival analysis showed the patients in the low-iAge subtype had significantly better overall survival (OS) than those in the high-iAge subtype (*p* < 0.001). In addition, iAge outperformed the chronological age in revealing the correlations with stem cell stemness. External validation demonstrated that iAge was an excellent method to classify cancer subtypes and predict survival in patients with GBM.

**Conclusions:** Inflammatory aging clock may be involved in the GBM *via* potential influences on immune-related activities. iAge could be used as biomarkers for predicting the OS and monitoring the stem cell.

## 1 Introduction

Glioblastoma (GBM) is the most common primary malignant tumor in adults, accounting for 48% of primary malignant tumors in the central nervous system (CNS) (Batash et al., 2017). Although the multidisciplinary treatments were taken, the median survival of GBM is merely approximately 15 months (Alifieris and Trafalis, 2015). The development of GBM is an evolutionary biological procedure with sequential steps, including genetic alteration, abnormal immunological functions, chronic inflammation, etc. (Waters et al., 2019; Ruff et al., 2020). It’s reported that the incidence of GBM rises dramatically after 54 years old and reaches a peak at the age of 74–85 years old (Ostrom et al., 2015). Several studies have identified that aging is a well-defined risk factor for GBM incidence and prognosis (Thakkar et al., 2014; Le Rhun et al., 2019).

Individual physical performance and health status markedly vary across chronological ages, at an accelerated or decelerated rate. It’s well appreciated that people at the same chronological age could have different physiological functions, while the individual at different chronological age may have same physiological conditions. Therefore, biological age, rather than chronological age, may be a more accurate biomarker to predict an individual age-related disorder. Aging is an inevitable time-dependent state and shares similar hallmarks with cancer (Aunan et al., 2017). One of the prominent features of aging is the stem cell exhaustion, which loss the capacity to maintain cellular homeostasis and repair injury (Rossi et al., 2008). Another hallmark in aging is the increase of low-grade inflammation, which is accompanied by the accumulation of proinflammatory damage and the dysfunction of immune cells (López-Otín et al., 2013). Contrary to acute inflammation, the aging cells secret diverse cytokines and induce the chronic adaptive immune response.

In 1863, Prof. Rudolf Virchow proposed the hypothesis that cancer may originate from chronic inflammation (Balkwill and Mantovani, 2001). Over the past century, the complex interplays between cancer and inflammation have been revealed. Nowadays, the mainstream view is that the GBM is cancer-induced inflammation, since the inflammation seems to be driven by oncogenes in GBM. In addition, radiation and cellular senescence could induce inflammation in GBM (Yeung et al., 2013). Cytokines released from inflammatory cells could promote tumor growth, enhance angiogenesis and induce metastasis (Coussens and Werb, 2002). Maybe the most meaningful clinical application is inflammation and anti-cancer therapy, and much data demonstrated that the anti-inflammatory drugs could reduce the tumor risk by 30% in some malignancies (Friis et al., 2015). Although the significances of inflammation are extensively explored, there are no standard methods to monitor the inflammatory cells changes and establish the reference values for age-related inflammation in GBM.

Recently, Prof. Furman David and his collaborative team have constructed a metric to estimate the age of systemic inflammation, termed inflammatory aging clock (Sayed et al., 2021). Similar to the epigenetic clock and transcriptomic clock, the inflammatory age (iAge) could track multifaceted aging phenotypes and have clinical significance in translation medicine. However, the potential applications of iAge in GBM have never been studied. Considering the intricated communications between inflammation and GBM, we systematically investigated the roles of iAge in GBM. We found that iAge was closely correlated with chronological age, and strongly correlated with inflammatory cell responses. Survival analysis showed that iAge could serve as a prognostic biomarker for overall survival (OS). Furthermore, the iAge could precisely predict GBM stem cells (GSC) stemness. Collectively, our findings provided evidence to depict the inflammatory clock, and characterize the iAge of patients with GBM.

## 2 Materials and methods

### 2.1 Acquisition of inflammation-related gene expression and clinical information

Inflammation-related genes (IRG) expression profiles were downloaded from the Cancer Genome Atlas (TCGA, https://portal.gdc.cancer.gov/), which contained the RNA-seq data and clinical information. The batch effect and normalization of the raw data in each sample were completed by the “sva” and “DESeq2” packages, respectively. We only recruited samples with complete RNA-seq data and clinical information, and excluded the patients with incomplete or unavailable information. The clinical information of patients with GBM included gender, age, tumor grade, TNM stage, OS, and survival status. In addition, patients with survival time less than 30 days were also excluded to reduce censored data and avoid survival bias. Two hundred IRG were retrieved from the previous study (Liang et al., 2021), and gene items were available in Supplementary Table S1.

### 2.2 Expression profiles of inflammation-related genes in pan-cancers and functional enrichment

The detailed summary of IRG was retrieved from the hallmark gene sets from the Molecular Signatures Database v7.4 (http://www.gsea-msigdb.org/gsea/msigdb/index.jsp),which contains 200 IFRGs. To investigate the IRG expression levels, we estimated the IRG expression profiles between tumors and corresponding normal samples in 20 cancers from TCGA. Next, significantly differentially expressed genes were compared between normal and tumor groups. Genes with |log2 fold change, logFC| > 1.5 and false discovery rate (FDR) <0.05 were considered significantly different.

To explore the IRG functions and possible mechanisms involved in the GBM, we performed the Gene Ontology (GO) and Kyoto Encyclopedia of Genes and Genomes (KEGG) enrichment analysis by the “clusterProfiler” package (Yu et al., 2012). Three levels annotations of KEGG were downloaded from KEGG Pathway Maps (https://www.kegg.jp/kegg-bin/get_htext?br08901.keg) (Shi et al., 2019). Then, to verify whether the pattern of differentially expressed genes appears randomly, we conducted the randomization test and repeated it 1,000 times. Next, to determine whether up- and downregulated IRG were significantly enriched in the inflammation-related activities, we performed the ROAST test by the “limma” and “statmod” packages. ROAST test is an alternative hypothesis that all target genes could be expected to regulate in the same direction (Wu et al., 2010).

#### 2.2.1 Data collection and curation of the inflammatory aging clock-related inflammation-related genes

According to age classification criteria proposed by Zhu et al. (2019), we divided chronological ages into four categories: the young group: ≤ 44 years old; the middle-aged group: 45–59 years old; the old-young group: 60–74 years old; old group: ≥75 years old. To identify inflammatory aging clock-related IRG, we calculated the correlation coefficients between IRG and chronological age in normal samples from pan-cancers. Then, the predicted iAge was calculated based on the formula: iAge = C * (β1 * X1 + β2 * X2 + β3 * X3 + ... βm * Xm). iAge is the predicted inflammatory age; C is the chronological age; β is the coefficient and X represents the IRG expression level.

#### 2.2.2 Characterization of glioblastoma subtypes and survival analysis based on inflammatory age

To predict the subtypes of patients with GBM, we divided patients into high- and low-iAge subtypes according to the median of iAge. Patients’ prognosis and survival status between high- and low-iAge subtypes were compared by the “reshape2”, “ggplot2” packages (Zhang et al., 2021).

To explore the survival values of the iAge-related IRG in patients with GBM, we first performed the univariate Cox regression analysis. Then, the random forest algorithm was applied to further screen the prognostic genes, and the seed was set as 12345678. The importance of each IRG, hazard ratio (HR), confidence interval (CI) and out-of-bag estimates were calculated. This work was done by the “randomForestSRC” package (Yuan et al., 2020).

### 2.3 Inflammatory age and stem cell index

To explore the associations between inflammation and stem cells in GBM, we analyzed the relationships between predicted age, chronological age and stem cell index using linear regression. The stem cell index included mRNA expression-based stemness index (mRNAsi), epigenetically regulated mRNAsi (EREG-mRNAsi), DNA methylation-based stemness index (mDNAsi), DNA methylation-based mDNAsi (EREG-mDNAsi) (Malta et al., 2018). Every stem cell index in each sample was obtained from the published study (Malta et al., 2018). The details of the stem cell index were provided in Supplementary Tables S2, S3.

### 2.4 External validation

**FIGURE 1:**
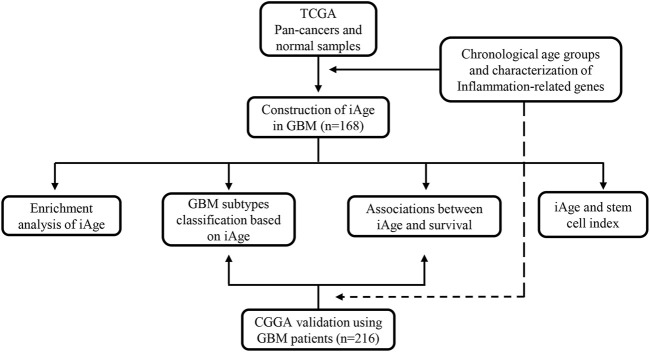
The flowchart of overall study design. Data acquisition and iAge establishment were based on TCGA dataset, and validation was from GEO database. TCGA, the Cancer Genome Atlas; iAge, inflammatory age; GBM, glioblastoma; CGGA, Chinese Glioma Genome Atlas.

We apply the Chinese Glioma Genome Atlas (CGGA) (http://www.cgga.org.cn/), a glioma-related resource with genomic and clinical data, to validate the results from TCGA. Patients were recruited for analysis according to the inclusion criteria: 1) primary GBM; 2) single GBM; 3) complete clinical information and genomic data. The external validation serves two purposes: 1) to verify whether iAge was an effective method to classify the GBM subtypes; 2) to verify whether iAge was a prognostic tool to predict patients’ survival. The overall study design is presented in Figure 1.

### 2.5 Statistical analysis

Wilcoxon matched-pair signed test was used to compare the gene expression differences between normal and 20 tumor samples. The statistical criteria of functional enrichment analysis were as follows: logFC > 1 and *p* < 0.05. Spearman correlation was used to correlation coefficients between IRG and chronological age, and the |coefficient| ≥ 0.5 was regarded as significant correlation. Cox regression was performed to select the prognostic factors. Log-rank test was applied to compare the survival differences, and the Kaplan-Meier curve was established to visualize the results. *p* < 0.05 was considered statistically significant. All the analyses were completed using R language software (version 4.0.3).

## 3 Results

### 3.1 Inflammation-related genes expression profiles in different chronological age groups

**FIGURE 2:**
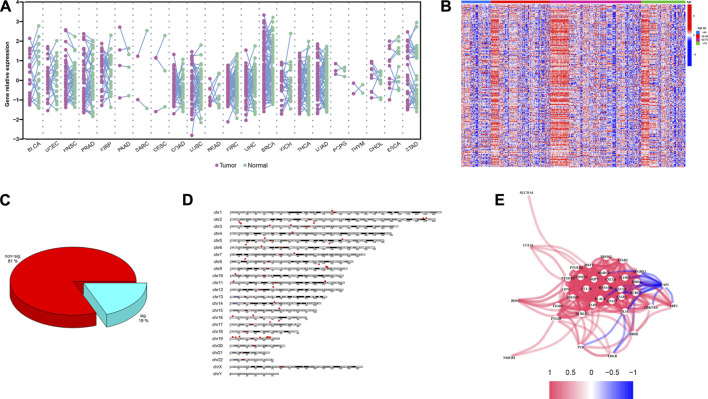
IRG expression profiles in pan-cancers and GBM. **(A)** The expression landscape of IRG in pan-cancers. Y axis represented the IRG relative expression level. Every purple dot indicated one tumor sample, while the normal sample in green. *p* values were calculated based on the two-tailed Student t-test. **(B)** Heatmap of IRG expression patterns in four different chronological age groups. Red represented the IRG high expression, while the low expression in blue. **(C)** Pie chart of the IRG. Spearman correlation showed 38 IRG (20%) were significantly associated with age (|coefficient| ≥ 0.5, *p* < 0.05). **(D)** Distribution of IRG on chromosomes. The 38 IRG were marked by red boxes. **(E)** Network analysis among 38 IRG. Red lines indicate the positive correlation, while negative in blue.

Chronological age refers to the length of time that one person has lived from birth to the time of calculation. To explore the expression profiles of the 200 IRG, we first collected the pan-cancers samples (*n* = 6249) and their corresponding normal samples (*n* = 702). The results showed the IRG expression levels were distinct between normal and tumor tissue in pan-cancer (Figure 2A). Then, we classified the normal samples (*n* = 702) into four groups according to the age classification criteria described above. The heatmap demonstrated the IRG expression pattern was significantly different among the four groups (Figure 2B). Next, we downloaded the GBM samples (*n* = 168) with complete clinical information and transcriptome data from TCGA according to the inclusion criteria, and we calculated the coefficients between IRG expression and chronological age in patients with GBM. The basic clinical information of patients with GBM was shown in Table 1. The results showed that there were 38 genes were significantly associated with chronological age (|coefficient| ≥ 0.5) (Figure 2C). The 38 IRG expression profiles in GBM samples were provided in Supplementary Table S4, and their correlations with age were provided in Supplementary Table S5. To further characterize the 38 IRG, gene positions in chromosomes were shown in Figure 2D. Next, we performed the network analysis among the 38 IRG, and the results were visualized in Figure 2E. MACRO, ADGRE1, IL15RA, RHOG, RNF144B, CXCL8, PLAUR, GNA15, C5AR1, CCRL2, PVR, LDLR, HRH1, IL18, CDKN1A, IRF1 and RAF1 had negative correlations. The other IRG had positive correlations.

**TABLE 1:** Basic clinical information of patients with GBM in TCGA (*n* = 168).

Characteristics	No.
Age (y)	59.23 ± 13.56
Gender	
Male	108 (64.29%)
Race	
White	149 (88.69%)
Karnofsky score	76.13 ± 14.69
Tumor longest dimension (cm)	0.77 ± 0.24
Survival status	
Dead	135 (80.36%)
Survival time (y)	1.98 ± 0.85

Data were presented in number (%) or mean ± SD.

### 3.2 Model construction of the inflammatory clock

**FIGURE 3:**
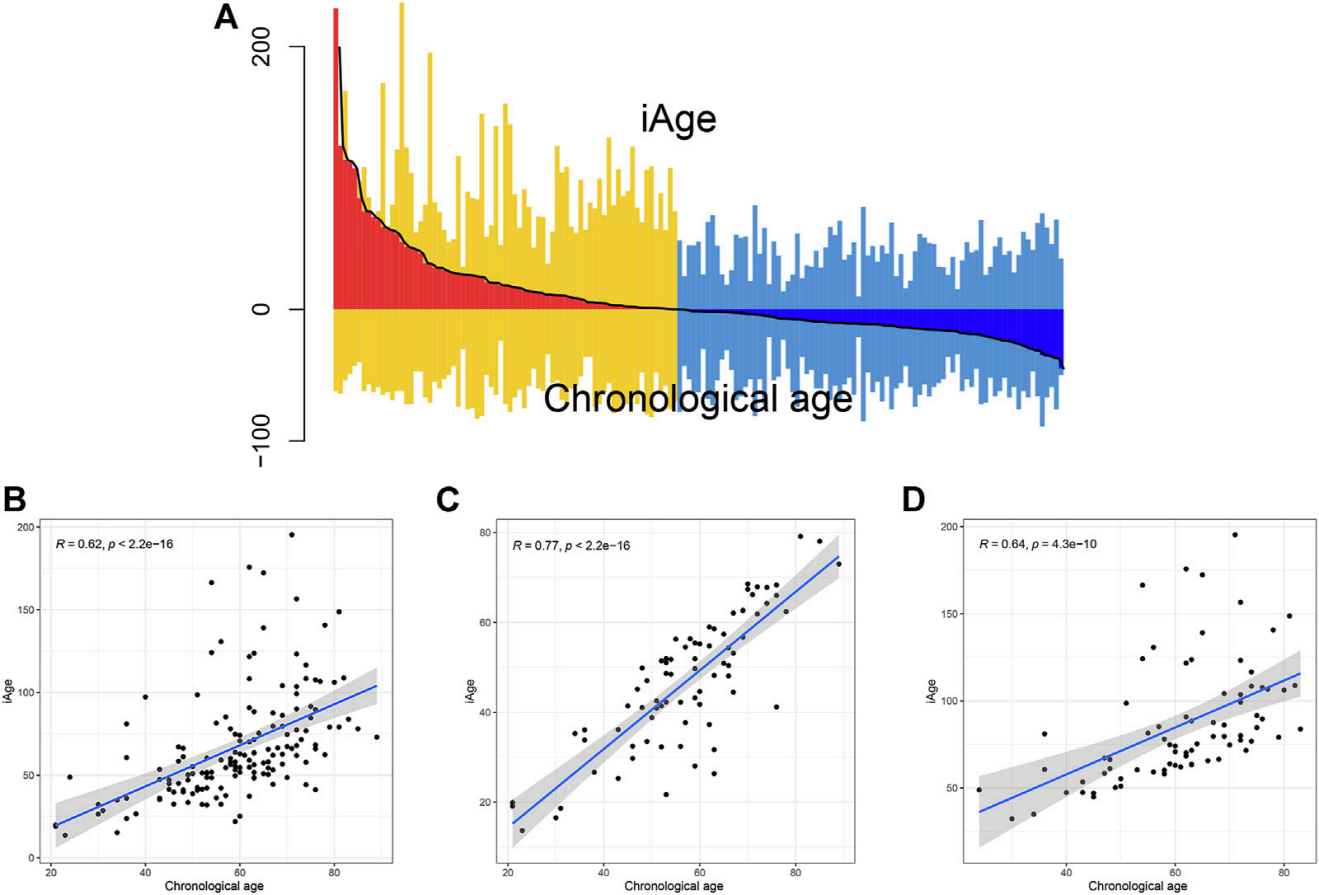
Correlations analysis between iAge and chronological age. **(A)** Differential pattern of iAge and chronological age. Above the X-axis is the iAge, and chronological ages below the X-axis. **(B)** Scatter diagram showed the correlations between iAge and chronological age in the entire cohort **(B)**, training cohort **(C)** and test cohort **(D)**.

The inflammatory aging clock was constructed to predict the inflammatory age based on the IRG expression levels and their correlations with chronological age. We controlled for iAge to ensure the authenticity and reliability of results. Through the linear regression, we found 38 IRG were significantly associated with chronological age (|coefficient| ≥ 0.5, *p* < 0.05), and used them to calculate the iAge according to the formula described above. The landscape of chronological age and iAge in patients with GBM was shown in Figure 3A. Linear regression demonstrated that they had a positive correlation (R = 0.62, *p* < 0.001) (Figure 3B). There were several abnormal prediction values (approximately 150 years old), suggesting the excessively activated inflammatory response. Next, we randomized the patients with GBM into training group and test group to evaluate the accuracy of the inflammatory aging clock. Training and test groups both exhibited excellent fitness and agreement (R = 0.77, *p* < 0.001; R = 0.64, *p* < 0.001) (Figures 3C,D, respectively). And the clinical characteristics of the training and test groups can be seen in Table 2.

**TABLE 2:** Basic clinical information of patients with GBM in training and test group.

Clinical characteristics	Training group (*n* = 84)	Test group (*n* = 84)	*p* value
Age (y)	57.14 ± 14.00	61.14 ± 12.97	0.39
iAge (y)	62.97 ± 15.69	70.19 ± 21.15	0.55
Gender			
Male	52 (61.90%)	56 (66.67%)	0.52
Race			
White	70 (83.33%)	79 (94.05)	0.03
Karnofsky score	77.38 ± 14.13	74.64 ± 15.49	0.19
Tumor longest dimension (cm)	0.75 ± 0.28	0.80 ± 0.18	0.06
Survival status			
Dead	60 (71.43%)	75 (89.29%)	0.004
Survival time	2.25 ± 0.79	1.76 ± 0.63	0.034

Data were presented in number (%) or mean ± SD.

### 3.3 Functional enrichment

**FIGURE 4:**
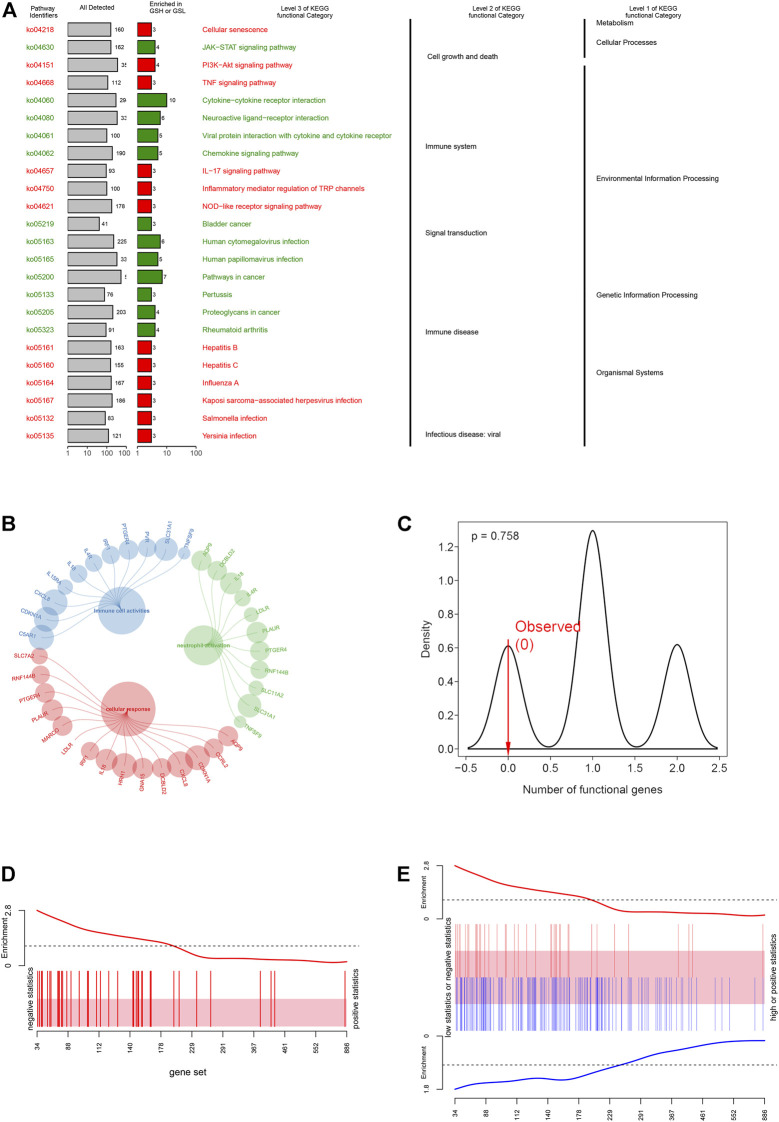
Functional enrichment analysis. **(A)** KEGG enrichment analysis showed the 38 IRG were strongly associated with cellular response and immunological activities. **(B)** GO enrichment analysis demonstrated that IRG had close relationships with inflammatory cell activation, cellular response and immune cell activities. **(C)** iAge-related IRG randomization test result. The *p* value implied the probability that iAge-related IRG are randomly involved in the inflammatory activities. Gene set bar **(D)** and barcode plot **(E)** analysis. iAge-related IRG were visualized as a shaded rectangle. The red bars represented the upregulated IRG, while the downregulated ones in blue. The enrichment scores above and below the shaded rectangle indicated the enrichment levels.

To explore the physiologies and functions in which iAge was involved, KEGG functional categories were performed. KEGG results showed that the 38 genes were enriched in cell growth, cell death, immune system, and immune diseases (Figure 4A). Further analysis showed that iAge was significantly enriched in cellular senescence, cytokine-cytokine receptor and signal pathways. Consistent with the KEGG, GO results found the 38 genes were strongly associated with cellular response and immunological activities (Figure 4B). The above results indicated that iAge was strongly correlated with cellular senescence, and immunological activities. To determine the significance of the iAge-related IRG classification, we performed the randomization test with 1000 iterations. The result showed there was 75.8% probability of appearing in the iAge-related pathways (Figure 4C). Then, we used the ROAST algorithm to identify whether the iAge-related IRG were significantly enriched in the inflammatory activities. The results demonstrated that iAge-related IRG were highly correlated with inflammation (Figure 4D). Furthermore, the gene set analysis revealed that the up- and downregulated genes with expression changes were strongly correlated with iAge (Figure 4E). In a nutshell, the iAge-related IRG have close relationships with inflammatory activities.

#### 3.3.1 Characterization of the glioblastoma subtypes based on inflammatory age

**FIGURE 5:**
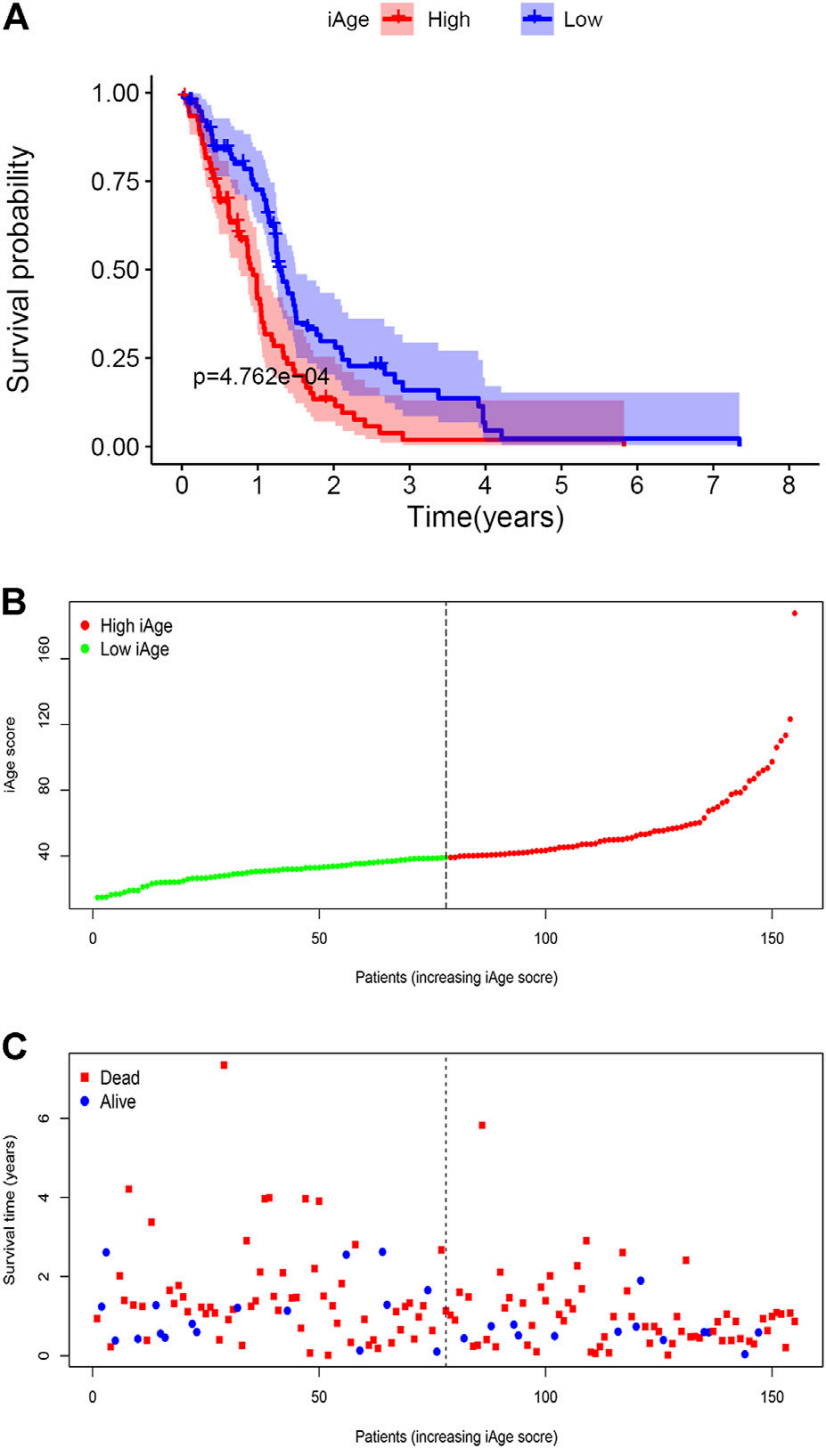
GBM subtype classification based on iAge. **(A)** Kaplan–Meier survival curve with log-rank test between high- and low-iAge subtypes. **(B)** Visualization of the iAge plot in high- and low-iAge subtypes. The inflection point refers to the median of iAge. **(C)** Survival distribution plot. The red dots represent the dead, and bule represent the alive.

We firstly calculated the iAge of each patient according to the above formula, and classified patients with GBM into high-iAge subtype (*n* = 84) and low-iAge subtype (*n* = 84). Then, we used three methods to evaluate the utilities of iAge clock: 1) Kaplan–Meier survival curve with the log-rank test between different subtypes; 2) iAge plot to visualize the predicted inflammatory age of each patient; 3) Survival status to illustrate patients’ outcomes. Our results showed that patients in the low-iAge subtype had significantly better OS than those with high-iAge (Figure 5A). Figure 5B demonstrated the iAge plot of each patient. With the increase of iAge, more and more patients died (Figure 5C). For these analyses, we controlled for age, Karnofsky score, gender, race and tumor size, because of the reported effect of each variable on the survival. Taken together, we successfully divided patients into high- and low-iAge subtypes.

### 3.4 Associations between inflammatory age and survival

**FIGURE 6:**
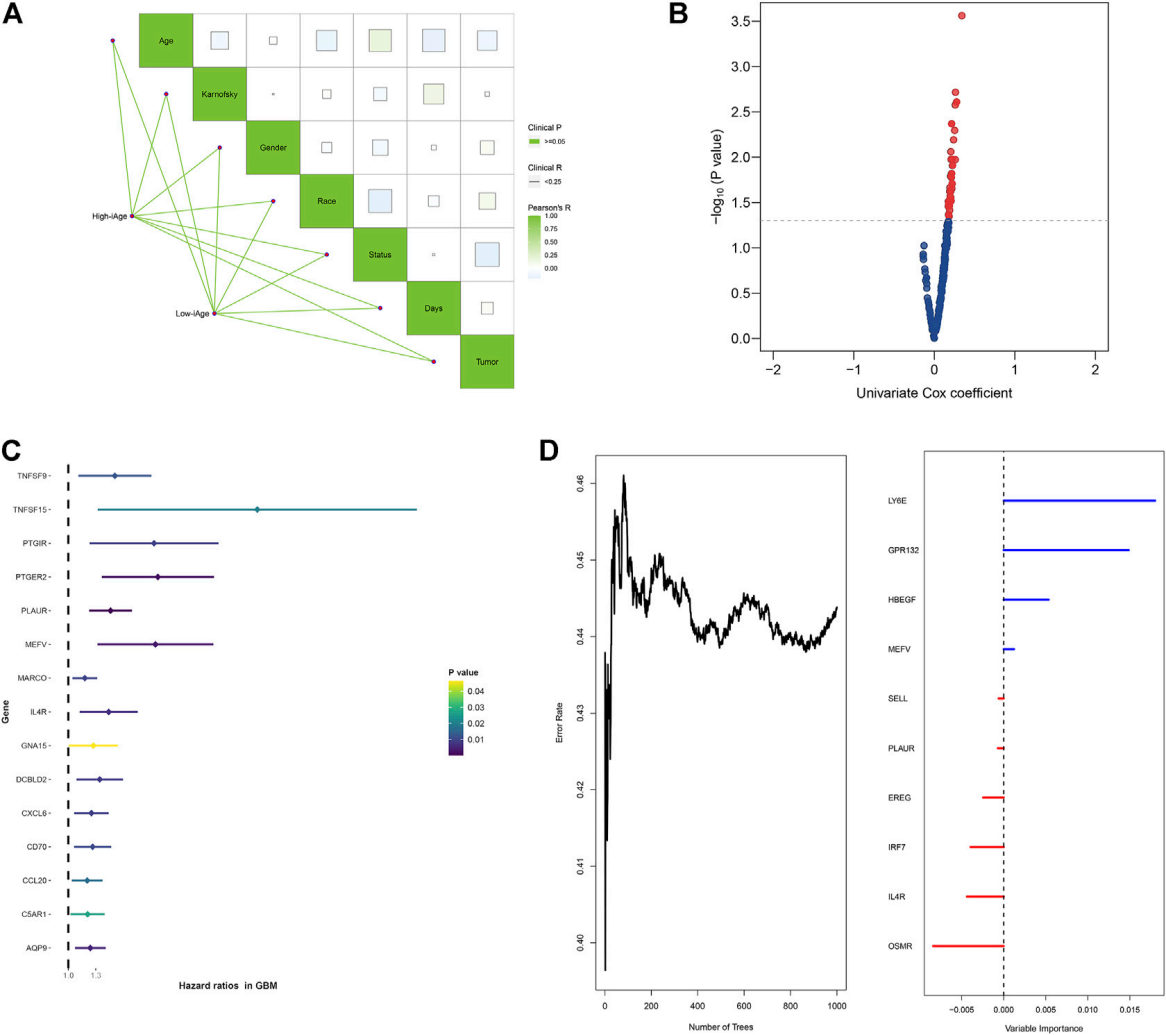
Survival analysis of IRG for OS in patients with GBM. **(A)** Correlations analysis between clinical information and subtypes groups. The data demonstrated there were no significant clinical differences between the two clusters. **(B)** Volcano plot demonstrated the significant prognostic genes with *p* < 005. **(C)** Forest plot showed the top 15 significant prognostic IRG. **(D)** Relations between number of trees and error rate, and the out-of-bag importance of each IRG.

To evaluate the influence of iAge on patients’ survival, we first investigated the correlations among the clinical features in the two GBM subtypes by the “ggcor” package. We observed there were no significant clinical differences between the two groups (Figure 6A). The univariate Cox regression results demonstrated there were 42 significant prognostic IRG for OS (*p* < 0.05) (Figure 6B). Figure 6C displayed the top 15 significant prognostic IRG (TNFSF9, TNFSF15, PTGIR, PTGER2, PLAUR, MEFV, MARCO, IL4R, GNA15, DCBLD2, CXCL6, CD70, CCL20, C5AR1, AQP9). They were all risk factors for OS (HR > 1). Next, the random forest was used for further selection. The relations between the number of trees and error rate were shown in Figure 6D (the left box). The gene importance was demonstrated in Figure 6D (the right box).

### 3.5 Correlations between the inflammatory age, chronological age and stem cell index

**FIGURE 7:**
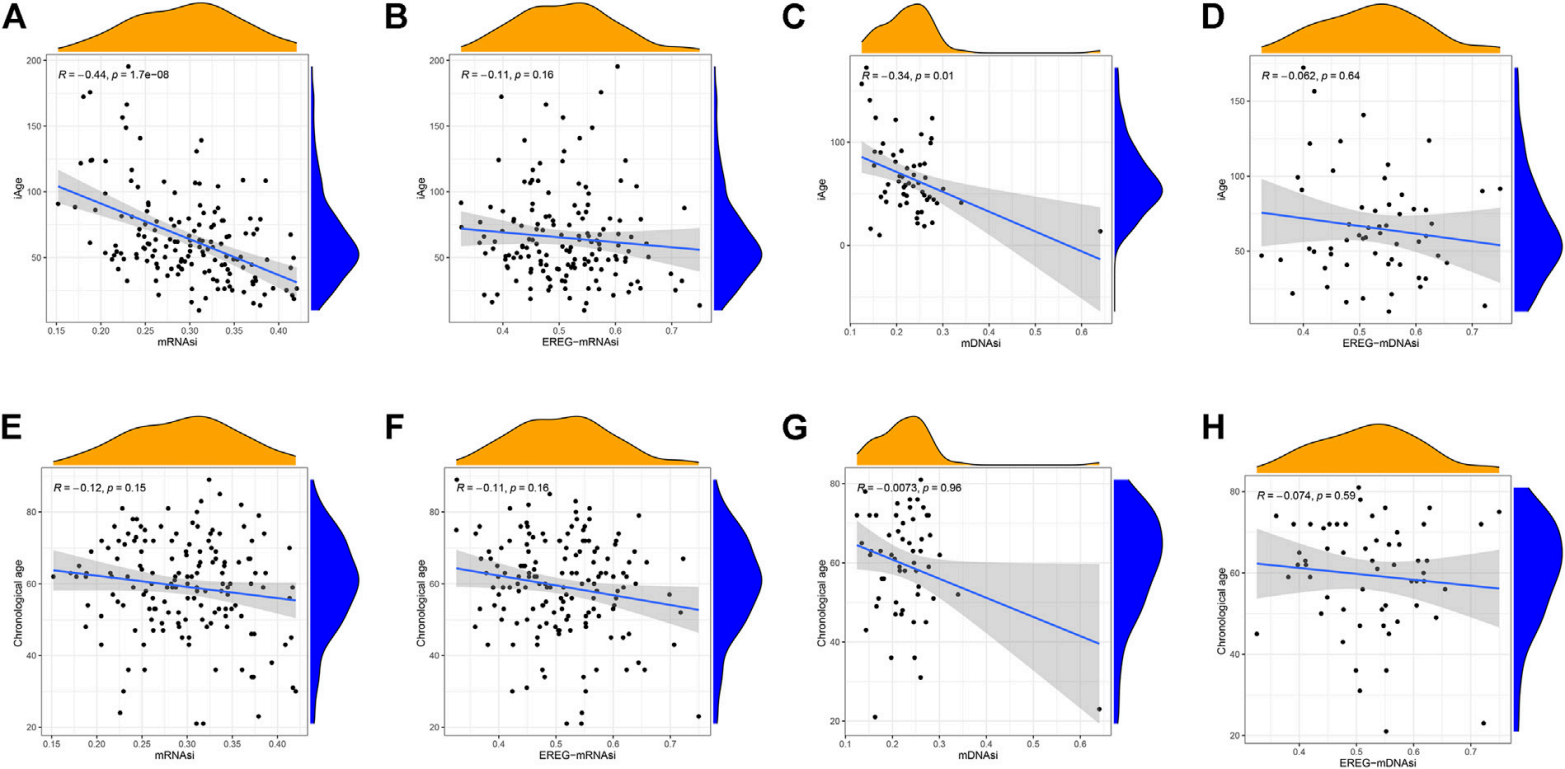
Correlation analysis between stem cell index, iAge, and chronological age. Correlation scatter plots depicting the relations between iAge and mRNAsi **(A)**, EREG-mRNAsi **(B)**, mDNAsi **(C)** and EREG-mDNAsi **(D)**. Similarly, Relations between chronological age and mRNAsi, EREG-mRNAsi, mDNAsi and EREG-mDNAsi were shown in **(E–H)**, respectively. *p* < 0.05 was considered as statistical significance.

Stem cell index is a comprehensive score describing the degree of similarity between cancer cells and stem cells. Thus, stem cell index can be regarded as the quantitative presentation of cancer stem cells. Age is a negative indicator of cell stemness, and a higher stem cell index implies a higher dedifferentiation ability and malignancy (Zhang et al., 2021). To explore whether iAge is a factor affecting stem cell differentiation, we calculated the correlations between prediction age, chronological age and four stem cell indexes (mRNAsi, EREG-mRNAsi, mDNAsi, EREG-mDNAsi). Our data demonstrated that iAge is a negative factor of mRNAsi (R = −0.44, *p* < 0.01) and mDNAsi (R = −0.34, *p* < 0.01) (Figures 7A,C, respectively), which generally indicated that iAge could serve as a useful biomarker to deduce the stem cells characteristics and help to understand the cancer progression. We observed no significant correlations between iAge, EREG-mRNAsi and EREG-mDNAsi (Figures 7B,D, respectively). The chronological age had no close relationships between mRNAsi, EREG-mRNAsi, mDNAsi, EREG-mDNAsi (|R|<0.2, *p* > 0.05) (Figures 7E–H, respectively). Collectively, our findings suggested that iAge is a better factor reflecting the GSC stemness superior to chronological age.

### 3.6 External validation of inflammatory age in CCGA

**FIGURE 8:**
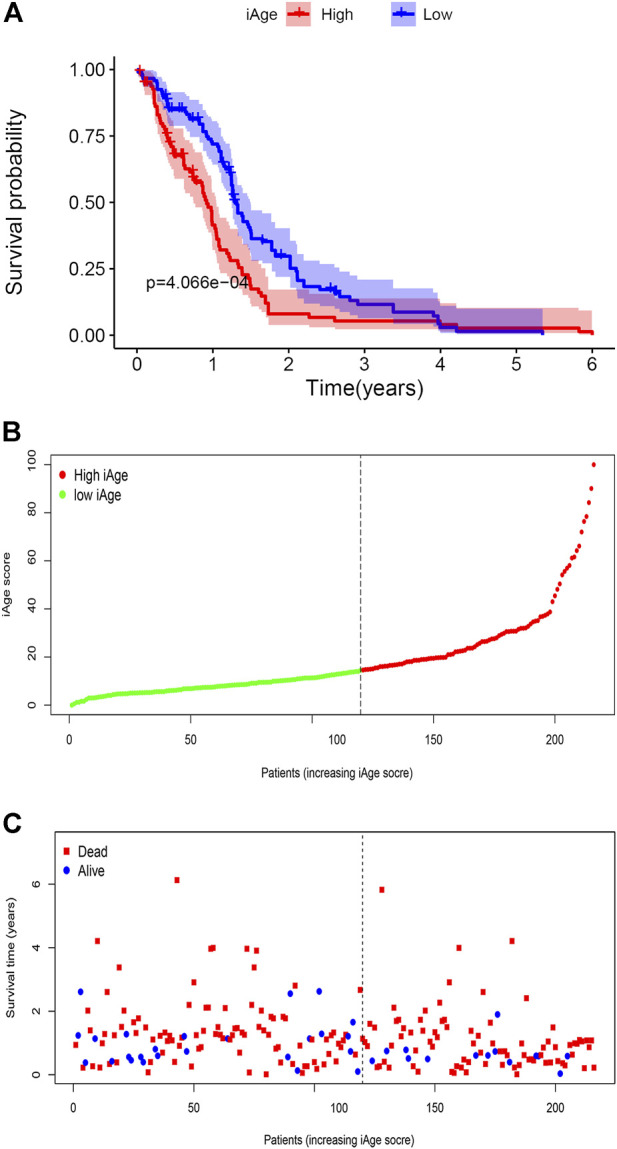
Validation of GBM subtype classification based on iAge from CGGA. **(A)** Kaplan–Meier survival curve with log-rank test between high- and low-iAge subtypes. Patients in the low-iAge subtype had higher probabilities to survive longer (*p* = 0.01). **(B)** Visualization of the iAge plot in high- and low-iAge subtypes. The inflection point refers to the median of iAge. **(C)** Survival distribution plot. The red dots represent the dead, and bule represent the alive.

A total of 388 patients with GBM were downloaded from CGGA. According to the inclusion criteria described above, we excluded 172 patients (*n* = 163 for recurrent and multiple GBM, *n* = 9 for unknown survival time). Finally, 216 patients with GBM were recruited for analysis. According to the original 38 IRG calculated in the TCGA, we used the same genes to construct the iAge in the CGGA (The expression data of the 38 genes were provided in Supplementary Table S6). Then, we calculated the iAge of each patient according to the above formula. To test whether iAge is an appropriate method to classify patients into different subtypes, we set the median of the iAge as the threshold to divide these 216 patients into high- and low-iAge subtypes (*n* = 108, 108, respectively). The data demonstrated that patients in the low-iAge subtype had significantly better OS than those in high-iAge subtype (Figure 8A). Figure 8B demonstrated the iAge distributions of each patient, and patients in different subtypes were marked with different color. In addition, with the increase of iAge, more and more patients died (Figure 8C). The available data implied iAge was a useful tool to classify GBM subtypes.

**FIGURE 9:**
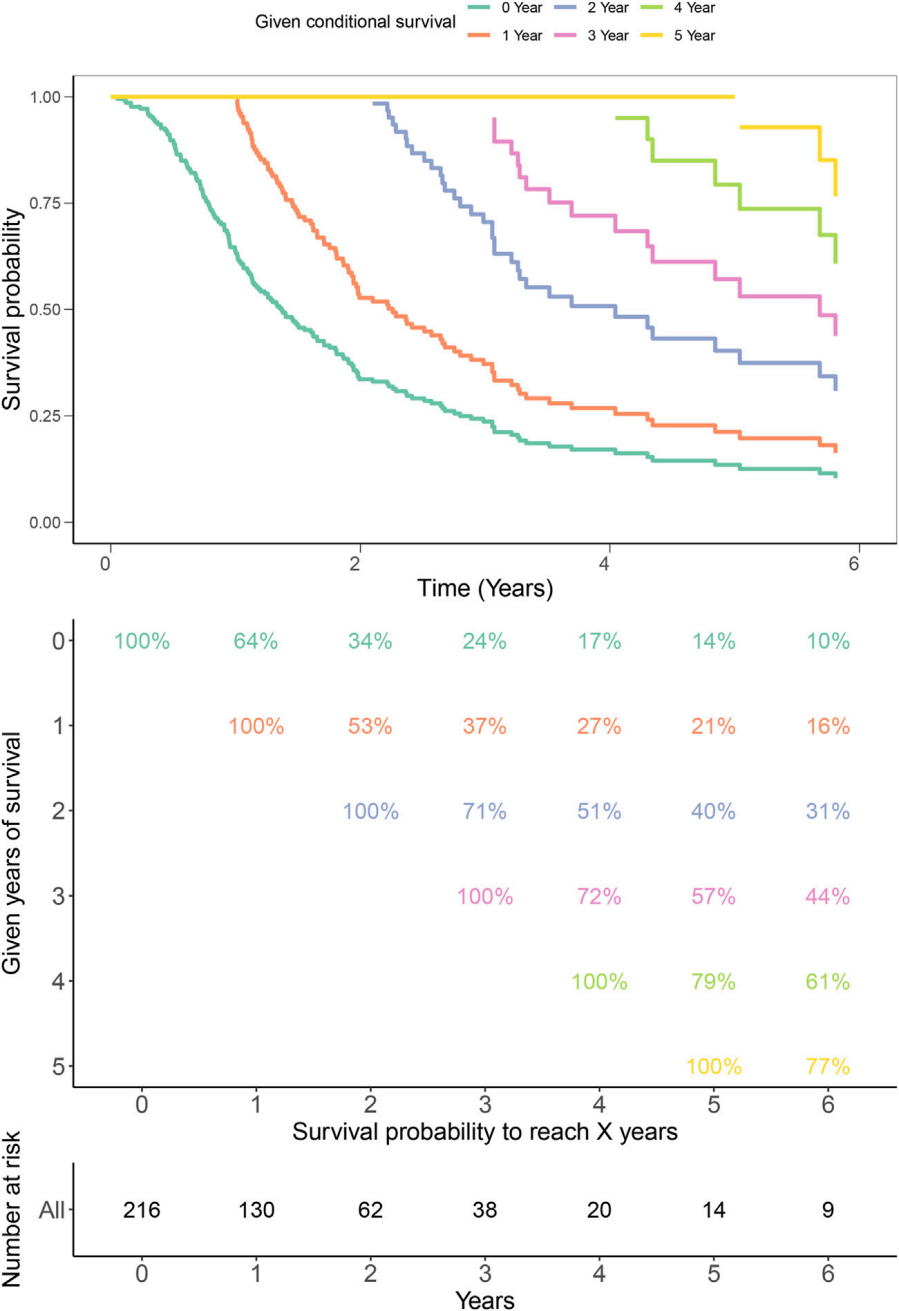
Kaplan–Meier estimates for conditional survival up to 6 years in 216 patients given 0–6 years’ survival. Every column indicated the survived year, and every row represented the survival percentage. The Kaplan-Meier curves were truncated at the maximal survival time (Upper). Survival probability table of each year was shown in numerical form (Middle). The number of total survived patients were represented in numerical form corresponding to the 0–6 years (Bottom). For example, if a patient has survived 3 years, the probability to be alive at 4th and 5th years is 72% and 57%, respectively.

Finally, to validate the prognostic values of iAge in patients with GBM, we performed the survival analysis and calculated the annual survival probability. The median overall survival was 1.26 years, with the 5-year survival rate of 15%. The survival probability decreased per year relative to the total survival time (Figure 9).

## 4 Discussion

Tumorigenesis is a multiple process involving a series of cellular changes that accumulate over time. Recent works revealed that some critical inflammatory components are dramatically elevated alone with age, especially in the tumors, such as GBM (Balkwill and Mantovani, 2001; Yeung et al., 2013; Kotas and Medzhitov, 2015). Thus, it’s reasonable to speculate that age played vital roles in inflammatory cells and the initiation of GBM. The latest study demonstrated that aging could be utilized to detect the inflammatory phenotypes in age-related disease (Sayed et al., 2021). To elucidate whether inflammation is influenced by age in GBM, we comprehensively explored the associations between age and inflammations through bioinformatics. In the present study, we found that the inflammatory aging clock was a reliable method to predict the inflammatory age and classify the GBM subtypes. Moreover, the GSC indexes decreased with the predicted inflammatory age. Functional enrichment analysis implied that the inflammatory aging clock was engaged in GBM by inflammation-related activities, immune cells, and immune response.

The proposed iAge model was constructed based on a similar algorithm that Horvath S adopted (Horvath, 2013). It’s optimal for the identification of the major inflammatory age contributors. Unlike other well-established cancer clocks (Horvath and Raj, 2018; Choukrallah et al., 2020), iAge is capable of capturing the inflammatory features, and strives to explain the complicated cross-talks between chronic inflammation and age. In this study, we found the iAge had the same tendency with the chronological age (R > 0.5), implying inflammatory dysfunctions are prone to accumulate when getting older. This result was accordant with the previous study, which pointed out that physiological aging usually brings with the activation of inflammatory signals and persistent inflammations (Franceschi and Campisi, 2014). More importantly, we applied the inflammatory clock to depict the differences between iAge and chronological age. The results unraveled that GBM is characterized by abnormal inflammatory expression patterns, and the age differences between iAge and chronological age could help to understand the GBM development.

Tumors with exactly the same histopathology may have completely different therapeutic responses and survival outcomes as a result of molecular heterogeneity. Current various subtypes have fully considered the key molecules that drive GBM progression, but failed to help improve survival (Lee et al., 2018). Owing to the large-scale studies emphasizing the significances of genome and inflammation, thus, we tentatively classified the patients with GBM based on the inflammatory aging clock. The results showed our subtype was robust and had good discrimination ability to cluster patients based on iAge. The subtypes classification derived from consensus clustering have aggregated multi-omics data, designated inflammation and survival information, and may successfully reflect the relations between GBM and inflammation. In addition, previous work revealed the roles of cancer clock in survival are context-dependent across different tumors (Lin and Wagner, 2015), suggesting the inflammatory clock may be a double-edged sword. In our study, survival analysis demonstrated that the inflammatory clock was a prognostic factor for OS, and could be utilized as an assessment tool for patients’ outcomes.

The inflammatory and immunological response failures to recognize and destroy malignant cells may be a result of the initiation of GBM. Our data revealed significant master genes of IRG in GBM. In accordance with previous studies, IL4R was demonstrated to be a useful biomarker for inflammation, and immunotherapy response (Puri, 1999; Ellingson et al., 2021). Similarly, CD70 was an essential component maintaining aggressiveness and recurrence, as well as inflammation and immune, suggesting IRG could help to unlock the resident GBM microenvironment (Seyfrid et al., 2022). In addition, IRG could act as promising biomarkers to predict prognosis in patients with GBM (Qiu et al., 2020; Seyfrid et al., 2022). Our study appears to be a novel result revealing the inflammatory presence and serve as potential biomarkers to predict survival.

GSC are responsible for therapeutic resistance and tumor recurrence (Sharifzad et al., 2019). Tumor progression is usually accompanied by the loss of cell differentiation and the acquisition of stemness. The regulation of GSC stemness involves intrinsic and extrinsic mechanisms, such as the immune system and tumor microenvironment (Lathia et al., 2015). Inflammatory cells inside the microenvironment could lead to an immunosuppressive situation that favors tumor growths (Lathia et al., 2015). In this study, we found the GSC stemness decreased with the increase of age. The iAge led us to assess the relationships between cancer stem cells and GBM, and provided us a method to identify inflammatory factors that may influence cancer development based on iAge. Furthermore, it’s notable that the iAge outperformed the chronological age in revealing the relevance with stem cell stemness (Figures 6A,C,E,G), implying that GSC with low iAge harbor more substantial tumor-promoting properties. Since all the tissues and organs are derived from stem cells, confirmation of stem cell characteristics by iAge will reflect the individual physical conditions and disease development. These findings implicated that iAge may be an appropriate candidate to monitor the GSC stemness.

The strength of this study is that we explored the inflammatory aging clock in GBM for the first time with reliable and repeatable statistical methods, and validated in an external cohort. Undeniably, there are also several limitations. Firstly, the iAge clock proposed by Prof. Furman David et al. was mainly based on blood immunome (Sayed et al., 2021). It’s universally known that variation in gene expression is extensive among tissue specimens and blood. Differences of iAge sources between blood and tissue specimens are inevitable. While it’s reasonable to identify the causality between iAge and GBM in the context of transcriptome data from TCGA, the biological aging clock needs to be experimentally tested in further study. Secondly, despite the proven utilities of iAge to characterize the patients’ subtype and predict the survival by the theoretical algorithm, it may be not applied in the real-world study. Thirdly, the exact mechanisms between inflammatory aging clock and GBM are unclear, although enrichment analysis was performed and may not completely mirror the physiologies *in vivo*. Finally, the iAge does not apply to all types of cancers because of the tumor heterogeneities. Notwithstanding its limitations, this study shed light on the link between inflammation and age in GBM, and these shortcomings will be resolved if there were enough data from a real-world study.

In conclusion, we identified the physiological importance and function of the inflammatory aging clock in GBM, which can be used to predict survival and monitor the stem cell. We provided novel insights into how iAge is a critical event for the development of GBM. Elucidation of the relations between inflammation and age will ultimately aid in the creation of new therapy that targets GBM.

## Data Availability

All data were available in TCGA database (https://portal.gdc.cancer.gov) and CGGA database (http://www.cgga.org.cn/). All the data displayed in the present manuscript are available from the corresponding author upon reasonable request.

## Abbreviations

GBM, glioblastoma; CNS, central nervous system; iAge, inflammatory age; OS, overall survival; GSC, GBM stem cells; IRG, inflammation-related genes; TCGA, the Cancer Genome Atlas; logFC, log2 fold change; FDR, false discovery rate; GO, Gene Ontology; KEGG, Kyoto Encyclopedia of Genes and Genomes; HR, hazard ratio; CI, confidence interval; mRNAsi, mRNA expression-based stemness index; EREG-mRNAsi, epigenetically regulated mRNAsi; mDNAsi, DNA methylation-based stemness index; EREG-mDNAsi, DNA methylation-based mDNAsi; CGGA, Chinese Glioma Genome Atlas.
